# Supplementary material for: Mass wasting reveals ongoing asymmetric retreat of the martian north polar ice cap
Source: Nat Commun. 2025 Jan 18;16:820. doi: 10.1038/s41467-025-56018-2 (PMC11743132; doi:10.1038/s41467-025-56018-2)
Supplement: Supplementary file 1 — Supplementary Information [file 41467_2025_56018_MOESM1_ESM.pdf]

Supplementary Information

Mass wasting reveals ongoing asymmetric retreat of the martian north polar ice cap

Shu Su[1,*], Lida Fanara[2], Haifeng Xiao[1,3], Ernst Hauber[2], and Jürgen Oberst[1]

[1]Institute of Geodesy and Geoinformation Science, Technical University of Berlin, 10553 Berlin, Germany.

[2]Institute of Planetary Research, German Aerospace Center (DLR), 12489 Berlin, Germany.

[3]Instituto de Astrofísica de Andalucía (IAA-CSIC), 18008 Granada, Spain

[*]Corresponding author: Shu Su, shu.su@campus.tu-berlin.de

Summary

Supplementary information for this manuscript includes Supplementary Figs. 1–26, Supplementary Tables 1–3, and Supplementary Method.

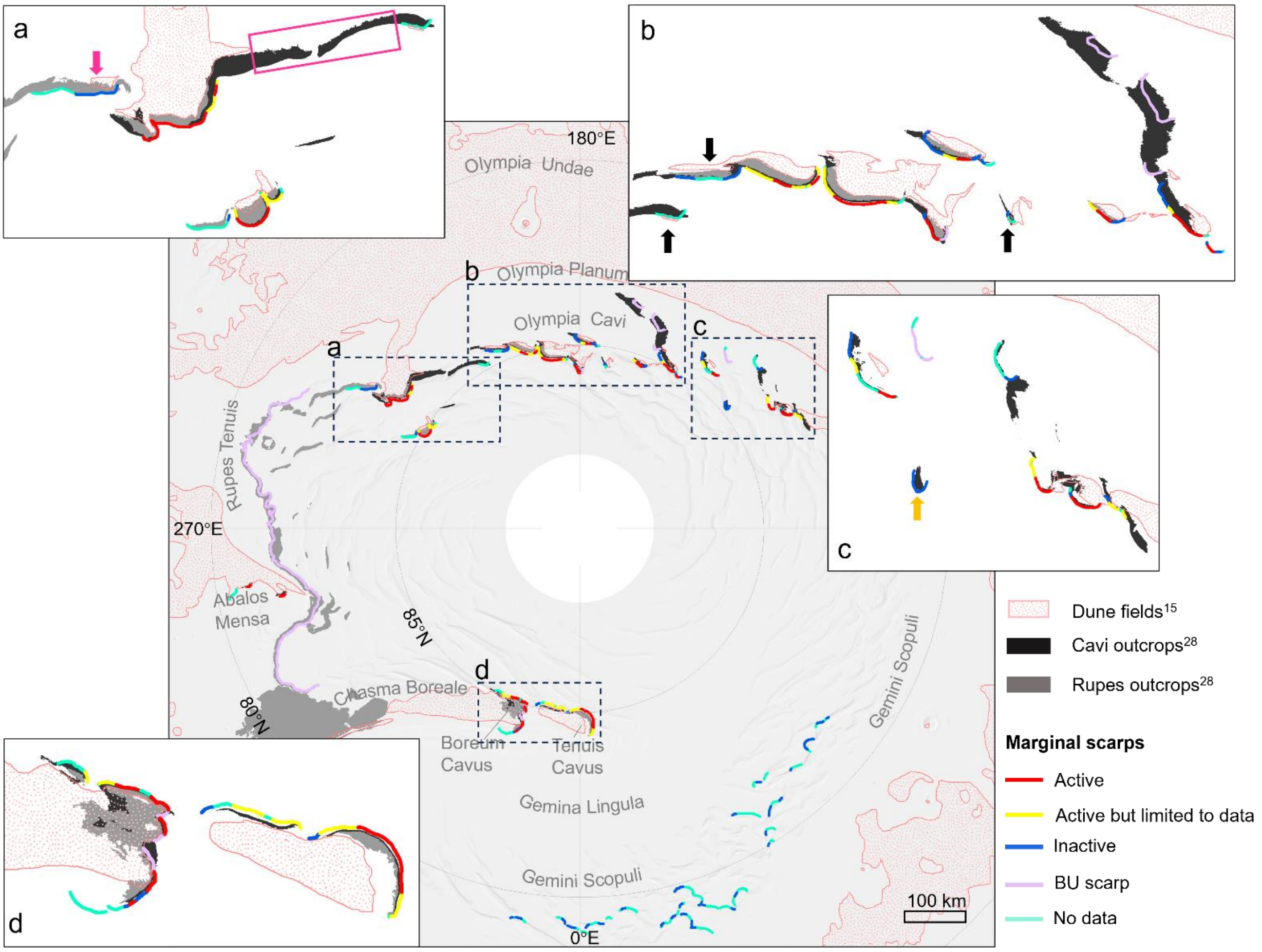

Supplementary Fig. 1 | Classification of marginal scarps based on ice block fall activities and available HiRISE data in northern summers from MY 28 to MY 36. We categorize the marginal scarps into 5 groups: the red lines depict the active scarps that have repeated HiRISE observations to perform change detection; the yellow lines depict the active scarps where the number of HiRISE images do not meet the criteria for producing Digital Terrain Models and/or applying change detection; the dark blue lines depict inactive scarps; the lilac lines depict the scarps in the BU region; the light green lines depict the scarps that have no HiRISE image from the northern summer time. The pink arrow in (a) points to an inactive NPLD scarp located directly above the rupes unit. The pink box in (a) outlines the regions with the cavi outcrops but no NPLD scarps. The black arrows in (b) point to the regions with both the BU outcrops and interior dune fields, while the NPLD scarps remain uncertain as to whether they are active due to lack of data. The orange arrow in (c) points to an inactive NPLD scarp located above the cavi outcrop. Dune fields are mapped by pink dots[15]. The cavi and rupes outcrops are mapped with black and grey patterns, respectively[28].

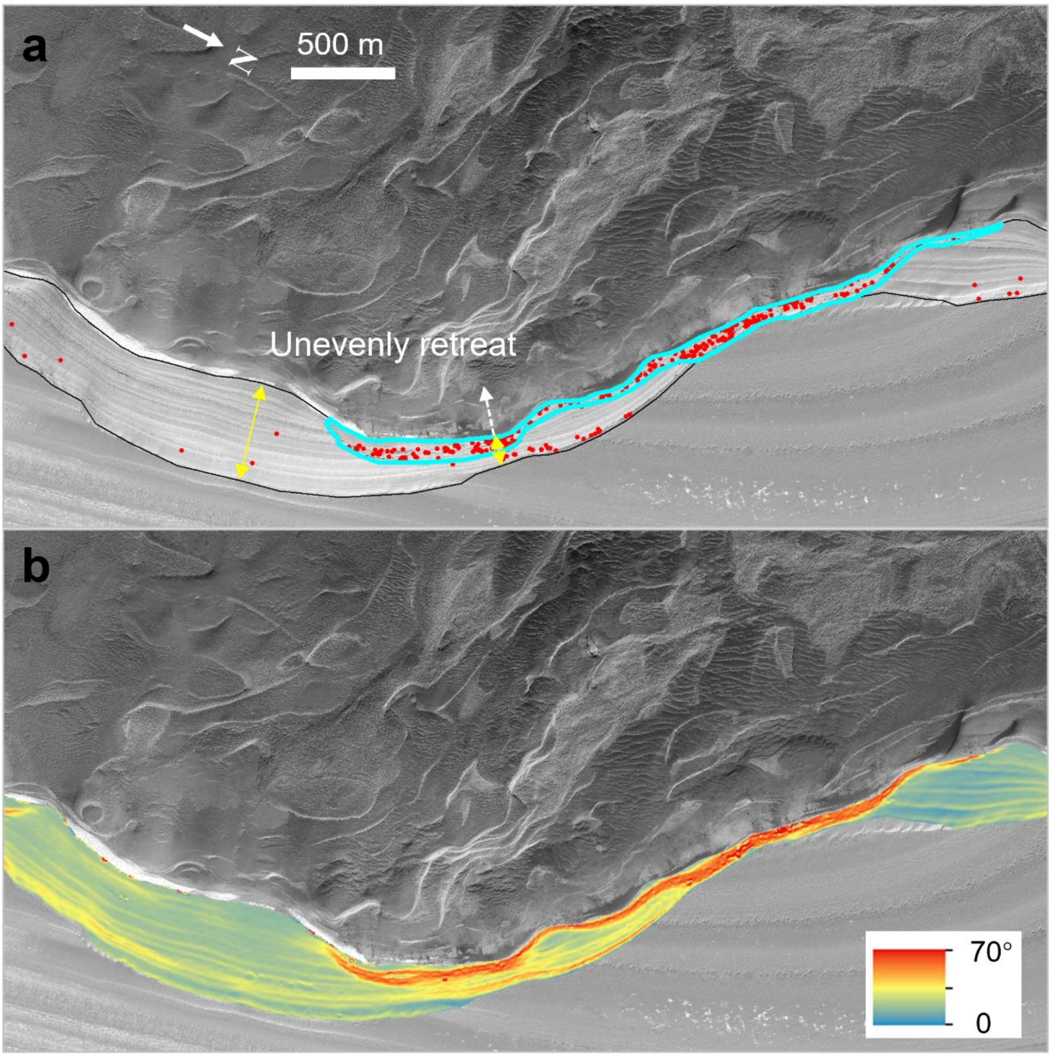

Supplementary Fig. 2 | Part of S1. a, Red dots correspond to the detached ice-fragments. The boundaries of S1 are depicted by black lines. The blue area highlights the active scarp part that shows the most detachments. The yellow arrows mark the unequal widths of the scarp, which indicate uneven retreat. **b**, Slope map of S1. The lower part of the scarp with the most detachments is the steepest part. Credit: background HiRISE image in **a** and **b**, NASA/JPL/University of Arizona.

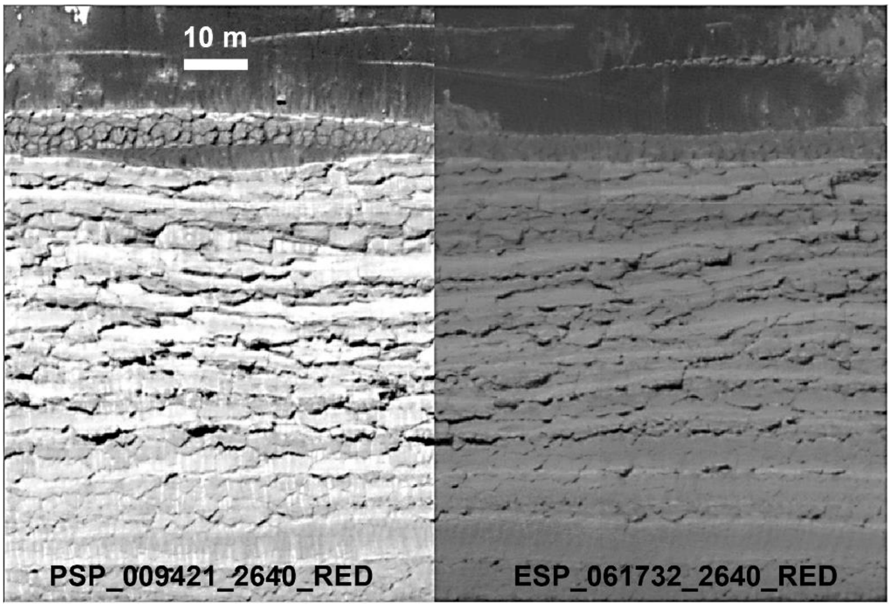

Supplementary Fig. 3 | Swipe view between orthorectified HiRISE images in MY 29 and in MY 35 at S2. The images are viewed in Esri's ArcGIS 10.3. Downhill is towards the top. Credit: background HiRISE image, NASA/JPL/University of Arizona.

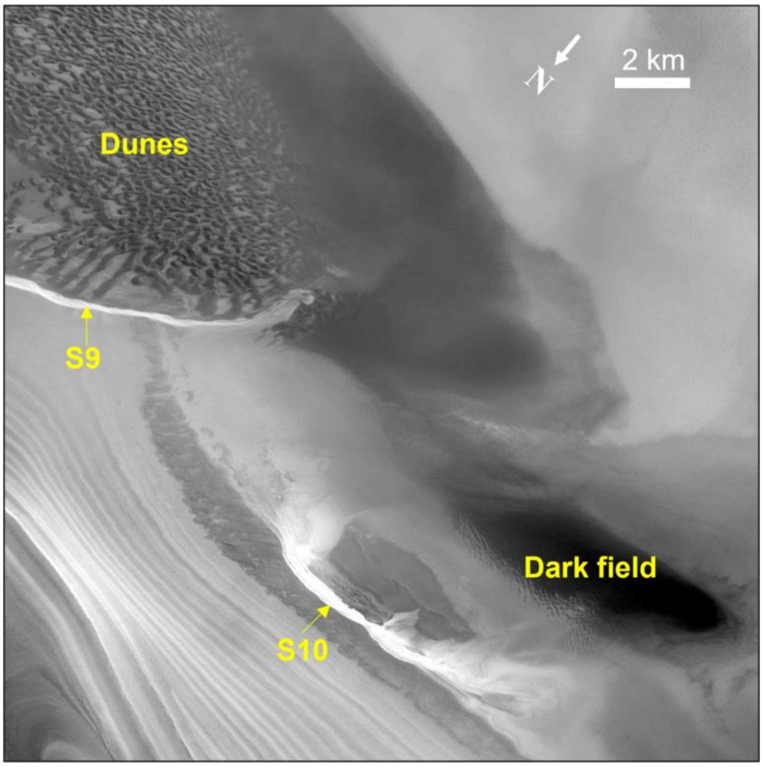

Supplementary Fig. 4 | A dark filed with similar albedo to the dunes near S9 lies downslope of S10. The background image is the MRO Context Camera (CTX) image G03_019472_2650_XN_85N212W. Credit: background CTX image, NASA/JPL-Caltech/MSSS.

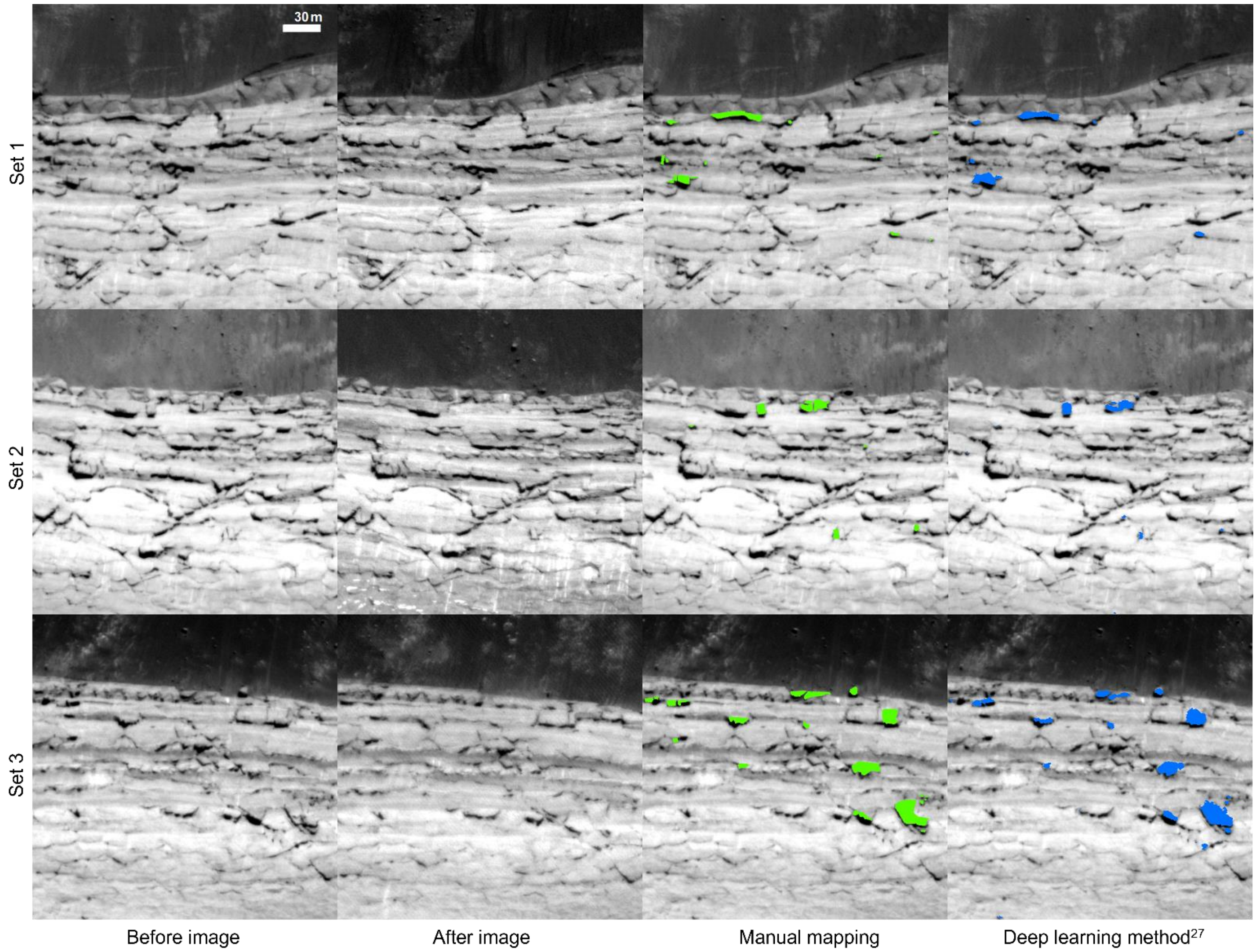

Supplementary Fig. 5 | Three sets of visualization results of detecting the detached ice-fragments by the deep learning

method[27] (blue) compared with manual mapping (green). For sets 1 and 2: the before image is part of HiRISE image PSP_009648_2650_RED in MY 29, and the after image is part of HiRISE image ESP_018905_2650_RED in MY 30. For set 3: the before image is part of HiRISE image PSP_009648_2650_RED in MY 29, and the after image is part of HiRISE image ESP_053730_2650_RED in MY 34. Credit: background HiRISE image, NASA/JPL/University of Arizona.

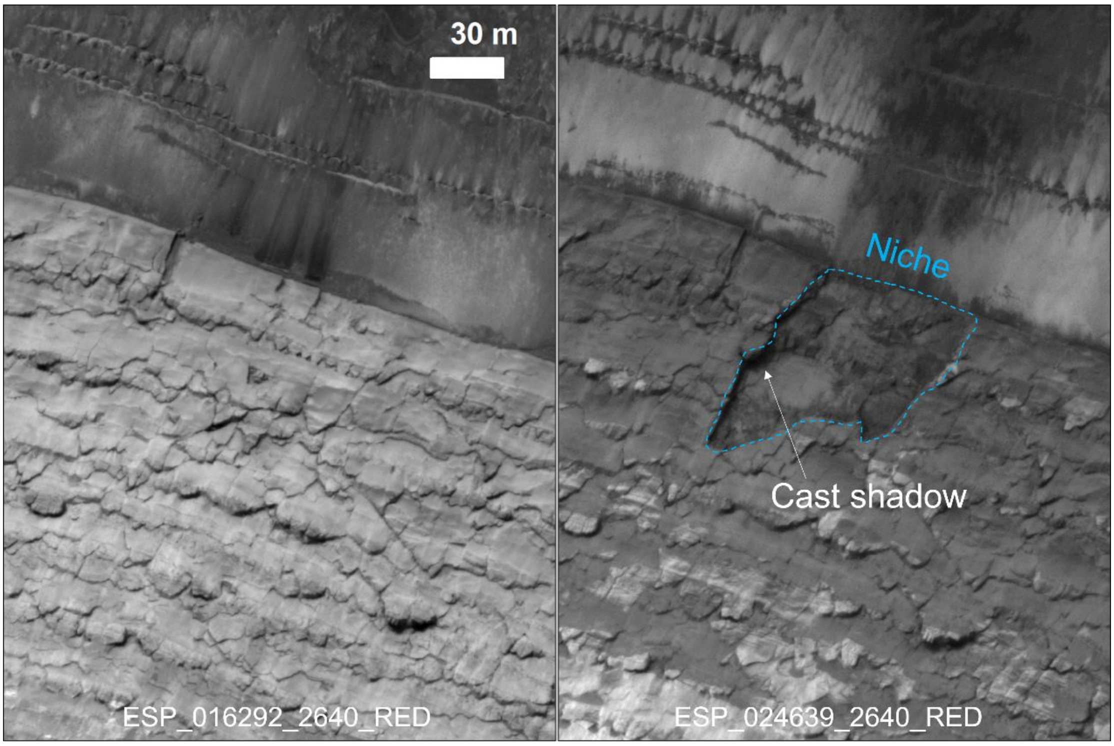

Supplementary Fig. 6| The before image (left) and after image (right) show the collapse of a slab-like ice-fragment. A niche, outlined by the blue dashed line, is shown in the after image. The shadow cast by the surrounding intact ice is pointed out by the white arrow. Credit: background HiRISE image, NASA/JPL/University of Arizona.

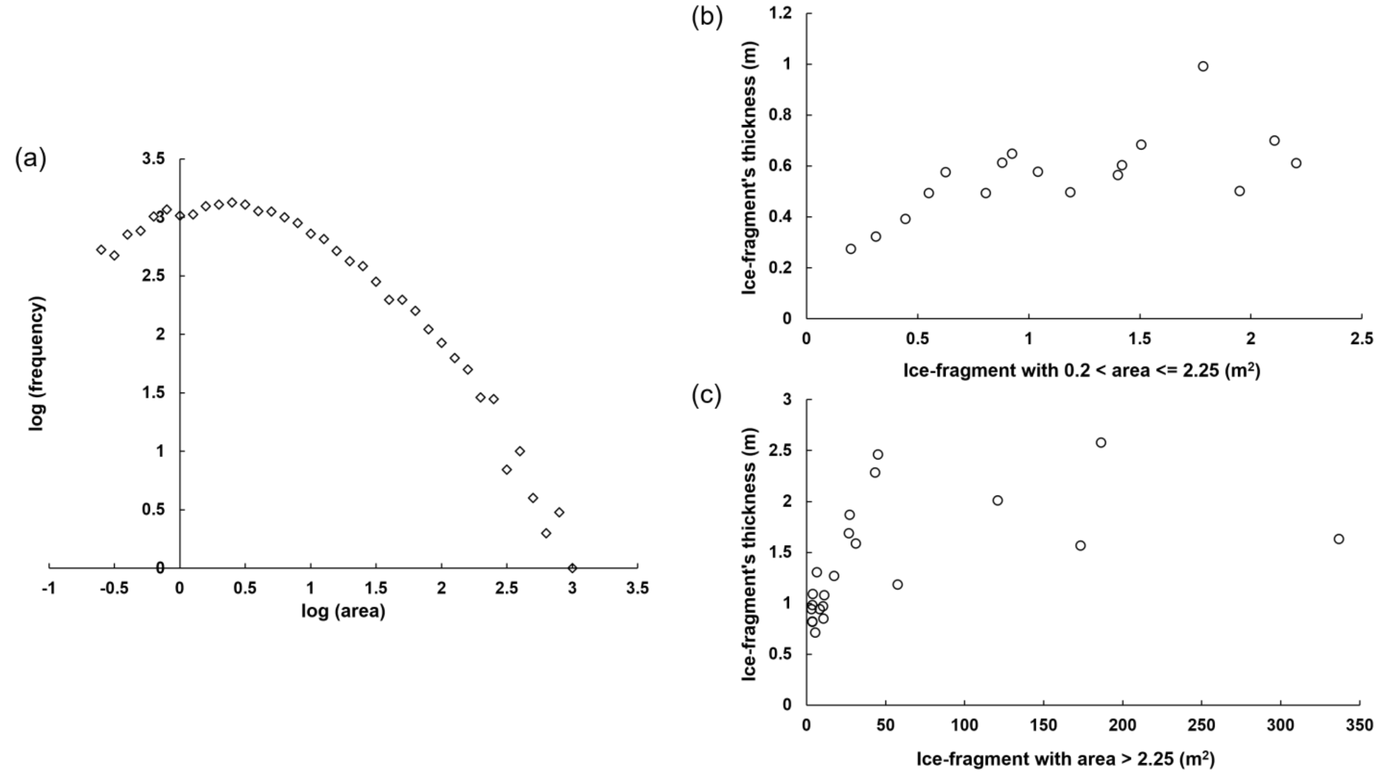

Supplementary Fig. 7 | a, The Log-log plot of size-frequency distribution of all detected ice-fragments. **b**, Thickness versus area for the selected 17 small-scale ice-fragments (0.2 m^2 < area <= 2.25 m^2). **c**, Thickness versus area for the selected 22 large ice-fragments (area > 2.25 m^2).

Supplementary Figs. 8-26 | Density maps of the ice-fragments' detection results on each scarp: S1–S19. Density is calculated as the number of detections in a circle with a radius of 100 m. The timeframe near the scarp indicates the time interval of the change detection. For example, MY 30–35 means the detection results are between MY 30 and MY 35. Note that one long scarp often needs more than one pair of change detection images because the size of the HiRISE image is not sufficient to cover the entire scarp. The corresponding HiRISE images for change detection are listed in Supplementary Table 3. The extents of the cavi outcrops, the rupes outcrops, and the dune fields are outlined in yellow.

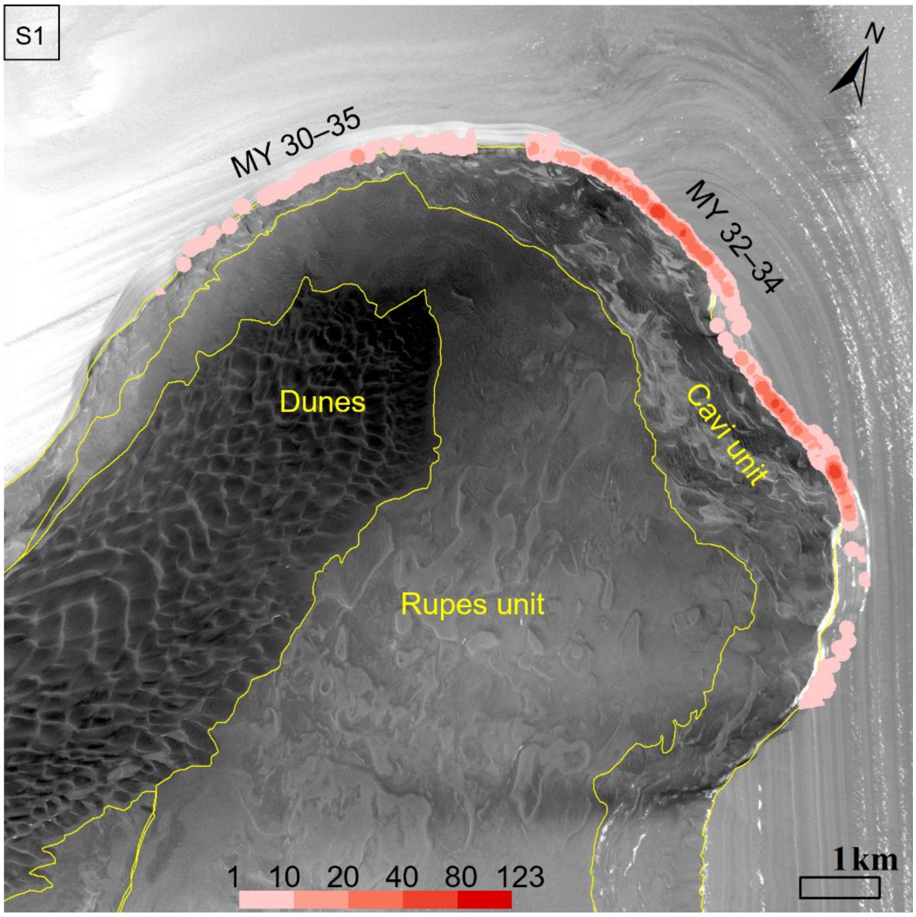

Supplementary Fig. 8 | Scarp S1. Downhill is towards the lower left. The background image is the Mars Reconnaissance Orbiter (MRO) Context Camera (CTX) image B21_017860_2640_XN_84N124W. Credit: background CTX image, NASA/JPL-Caltech/MSSS.

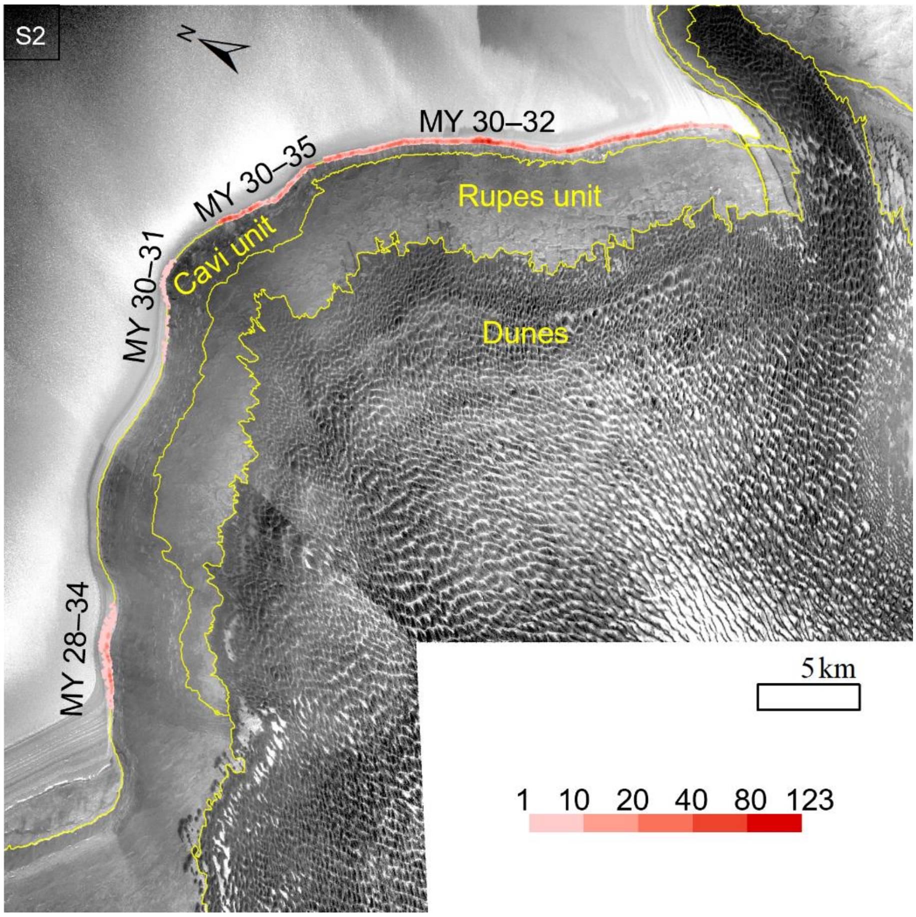

Supplementary Fig. 9 | Scarp S2. Downhill is towards the lower right. The background image is a mosaic of the CTX image B21_017900_2639_XN_83N126W and B22_018190_2641_XN_84N130W. Credit: background CTX image, NASA/JPL-Caltech/MSSS.

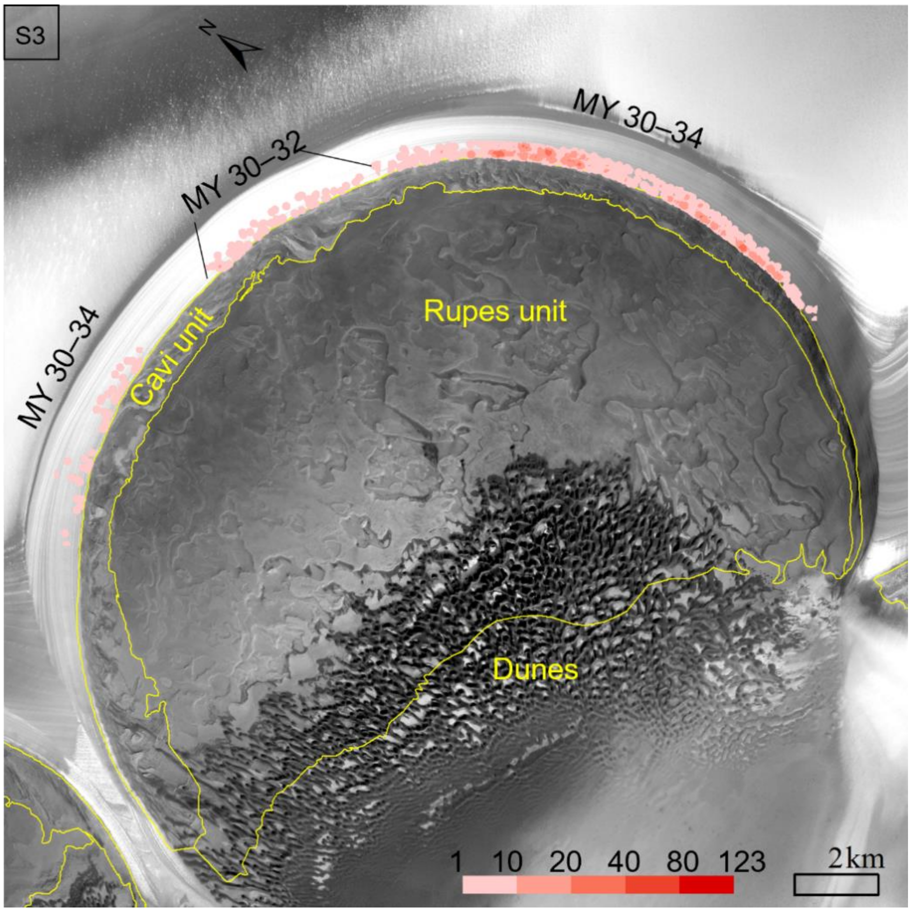

Supplementary Fig. 10 | Scarp S3. Downhill is towards the lower right. The background image is the CTX image D01_027776_2651_XN_85N121W. Credit: background CTX image, NASA/JPL-Caltech/MSSS.

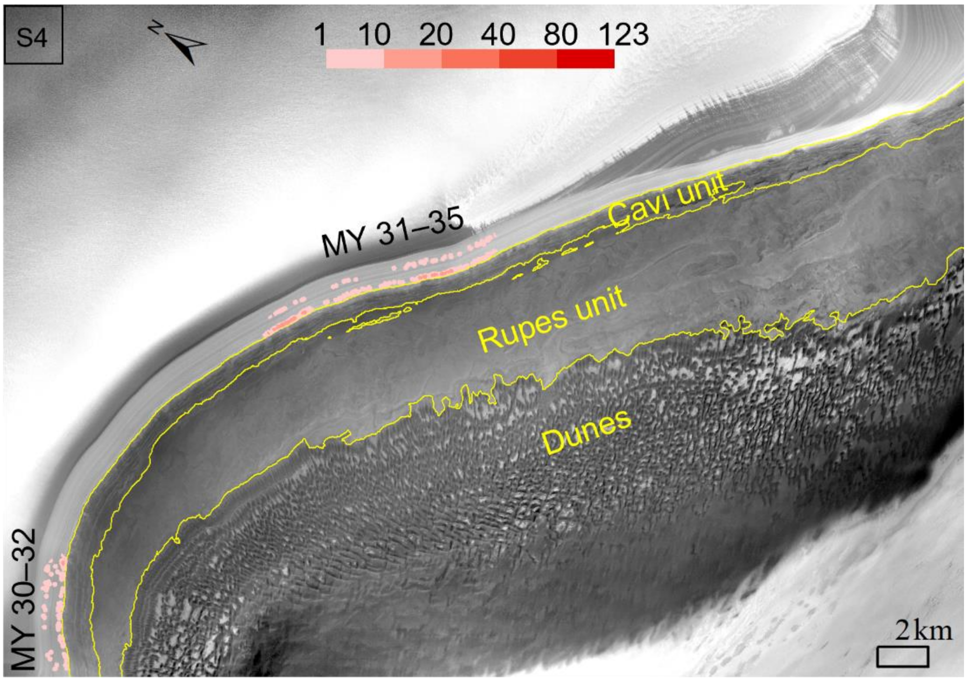

Supplementary Fig. 11 | Scarp S4. Downhill is towards the lower right. The background image is the CTX image D02_028015_2649_XN_84N161W. Credit: background CTX image, NASA/JPL-Caltech/MSSS.

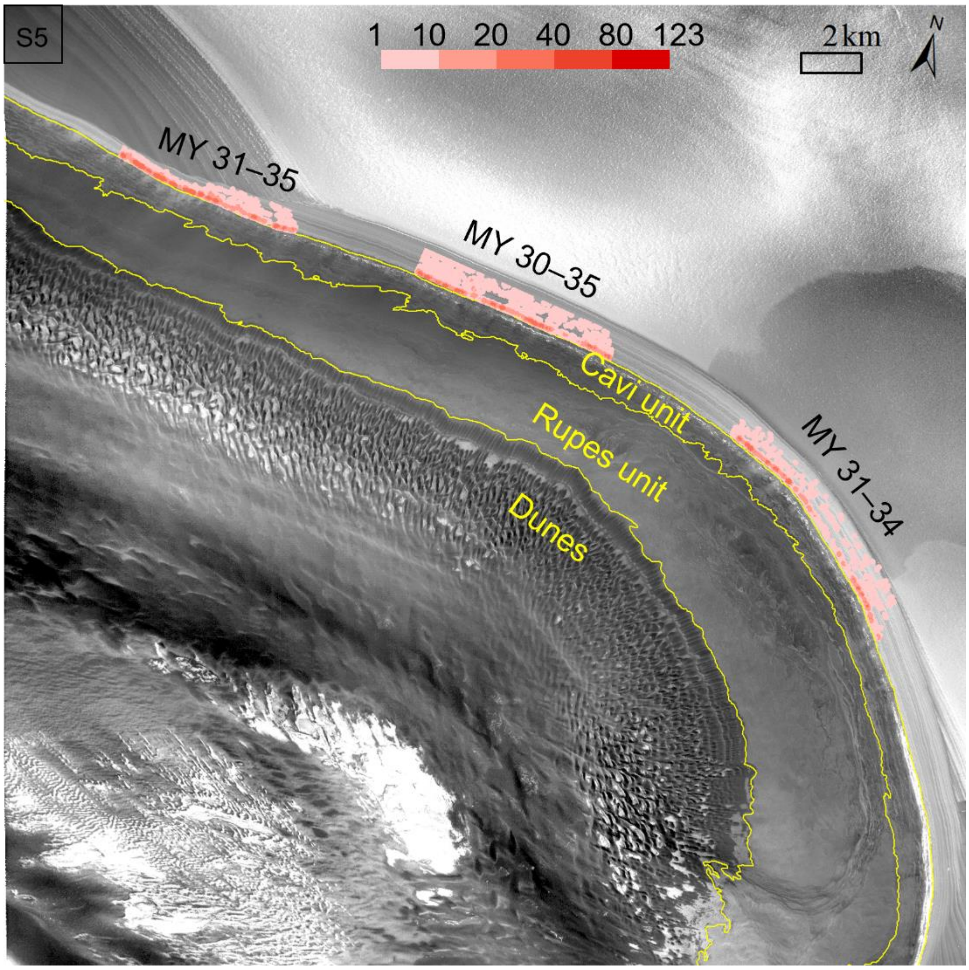

Supplementary Fig. 12 | Scarp S5. Downhill is towards the lower left. The background image is the CTX image B22_018403_2653_XN_85N170W. Credit: background CTX image, NASA/JPL-Caltech/MSSS.

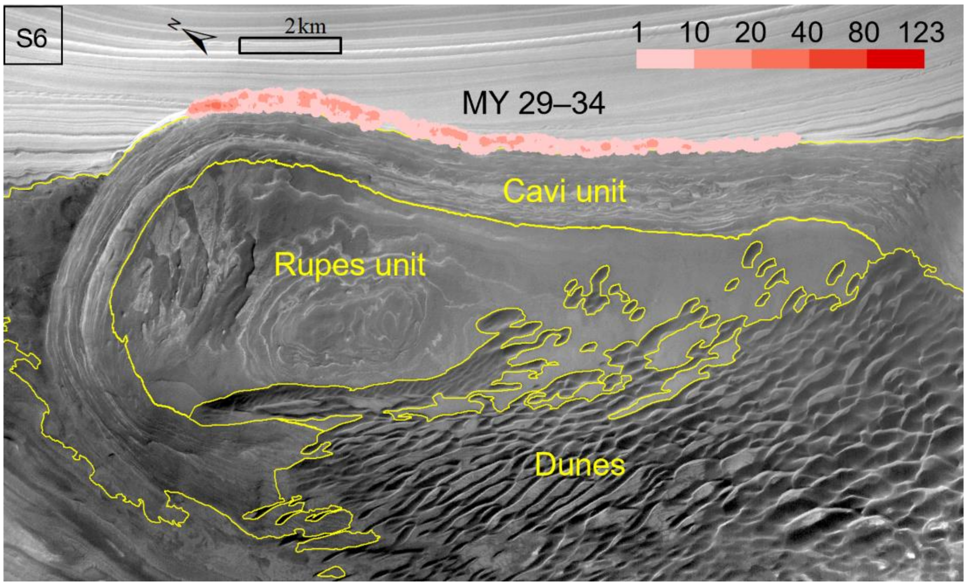

Supplementary Fig. 13 | Scarp S6. Downhill is towards the lower right. The background image is the CTX image B03_010636_2658_XN_85N180W. Credit: background CTX image, NASA/JPL-Caltech/MSSS.

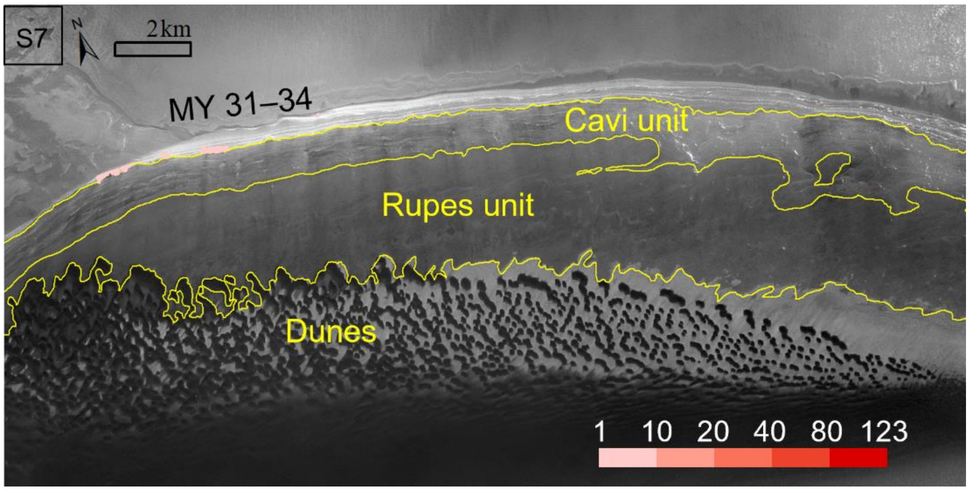

Supplementary Fig. 14 | Scarp S7. Downhill is towards the bottom. The background image is the CTX image G22_026631_2650_XN_85N182W. Credit: background CTX image, NASA/JPL-Caltech/MSSS.

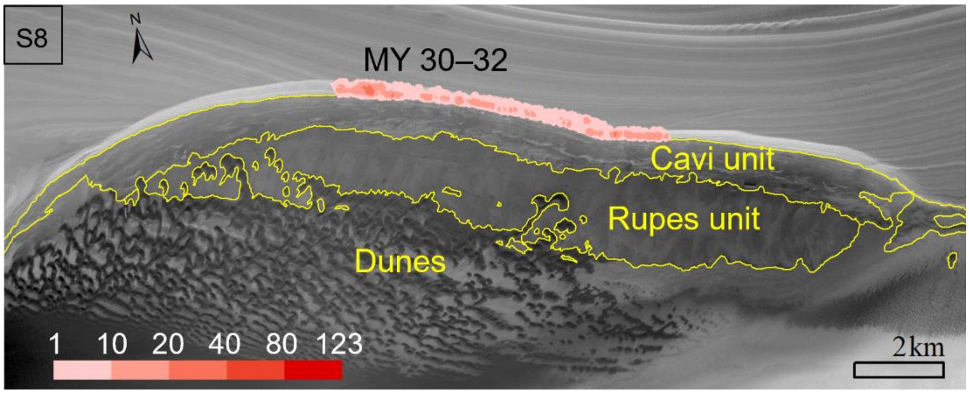

Supplementary Fig. 15 | Scarp S8. Downhill is towards the bottom. The background image is the CTX image D01_027647_2652_XN_85N199W. Credit: background CTX image, NASA/JPL-Caltech/MSSS.

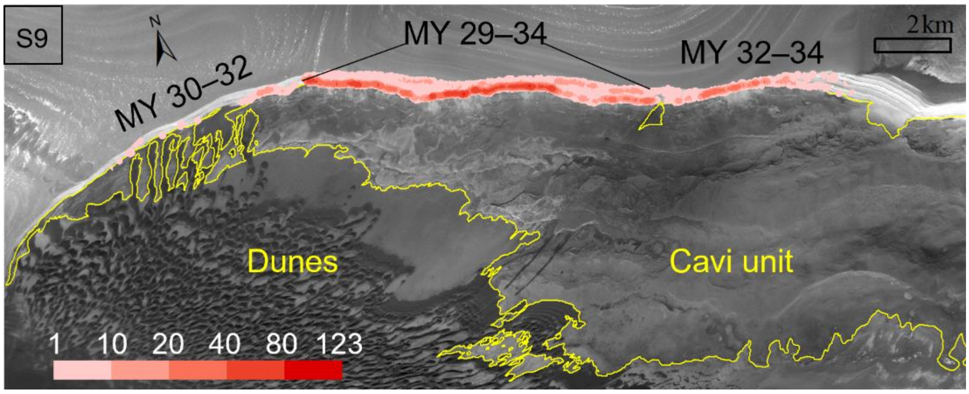

Supplementary Fig. 16 | Scarp S9. Downhill is towards the bottom. The background image is the CTX image B01_010097_2650_XN_85N209W. Credit: background CTX image, NASA/JPL-Caltech/MSSS.

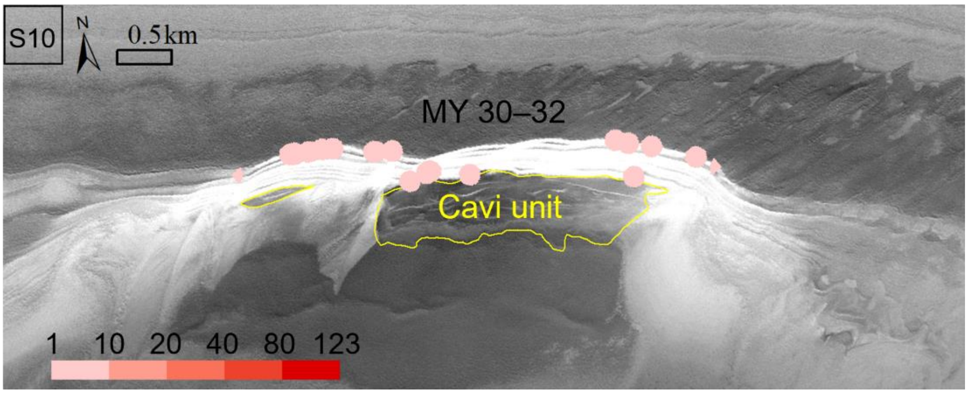

Supplementary Fig. 17 | Scarp S10. Downhill is towards the bottom. The background image is the CTX image G03_019472_2650_XN_85N212W. Credit: background CTX image, NASA/JPL-Caltech/MSSS.

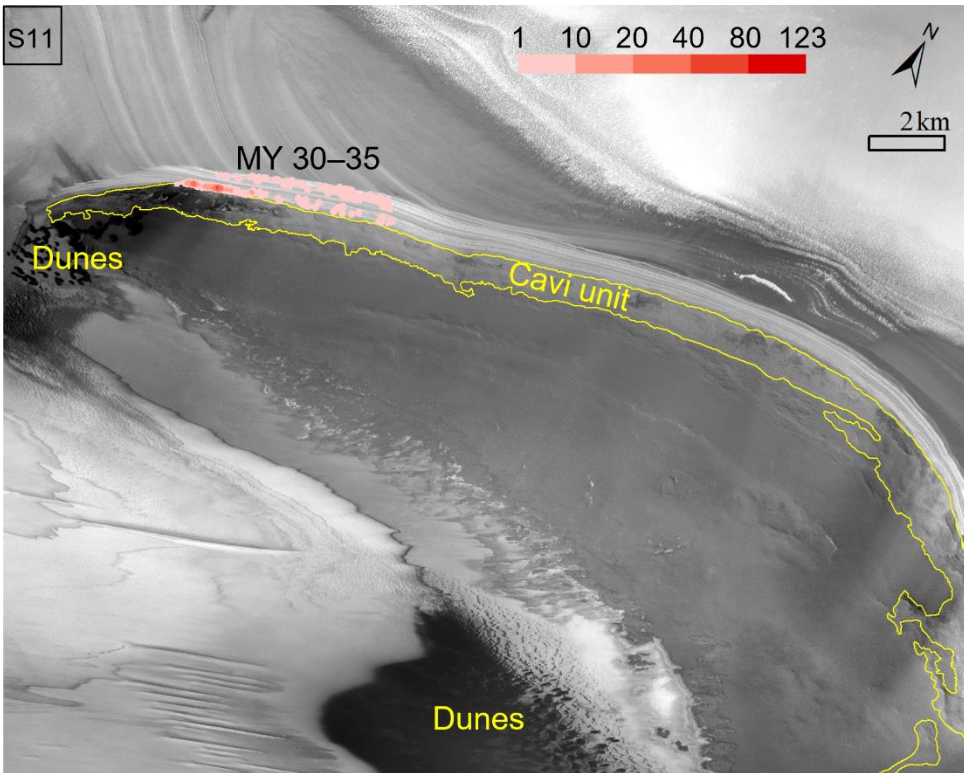

Supplementary Fig. 18 | Scarp S11. Downhill is towards the lower left. The background image is the CTX image B01_009886_2642_XN_84N219W. Credit: background CTX image, NASA/JPL-Caltech/MSSS.

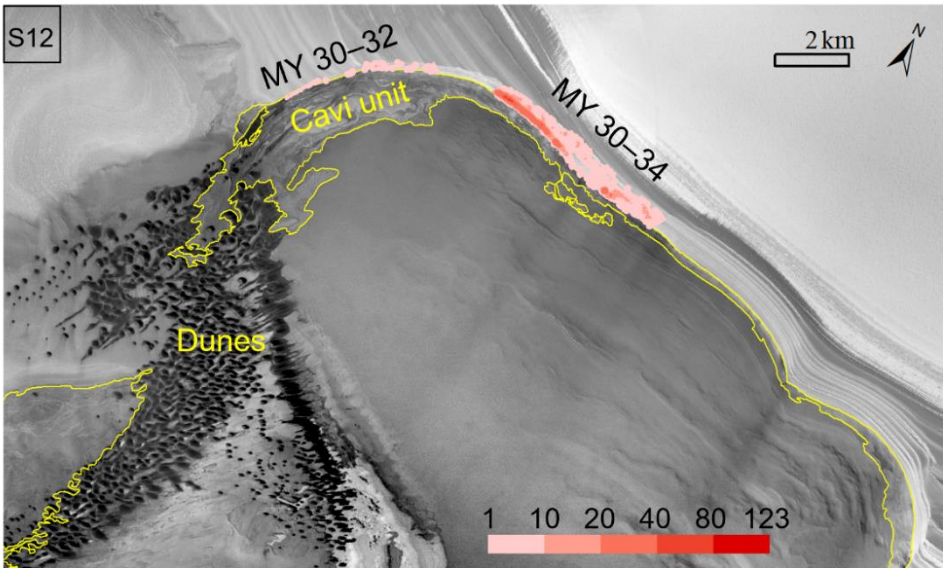

Supplementary Fig. 19 | Scarp S12. Downhill is towards the lower left. The background image is the CTX image D01_027675_2639_XN_83N237W. Credit: background CTX image, NASA/JPL-Caltech/MSSS.

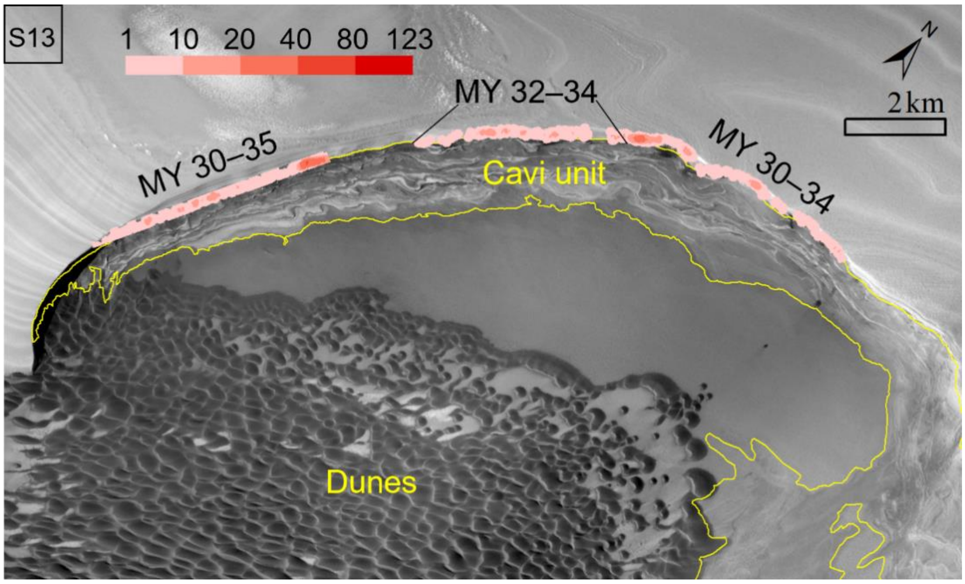

Supplementary Fig. 20 | Scarp S13. Downhill is towards the lower left. The background image is the CTX image D01_027662_2636_XN_83N239W. Credit: background CTX image, NASA/JPL-Caltech/MSSS.

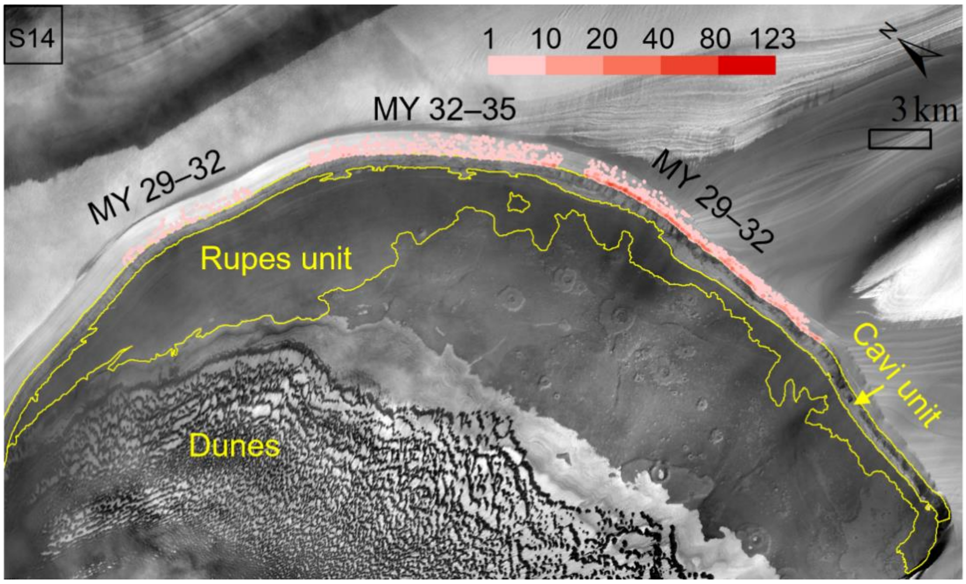

Supplementary Fig. 21 | Scarp S14. Downhill is towards the lower left. The background image is the CTX image D01_027745_2649_XN_84N357W. Credit: background CTX image, NASA/JPL-Caltech/MSSS.

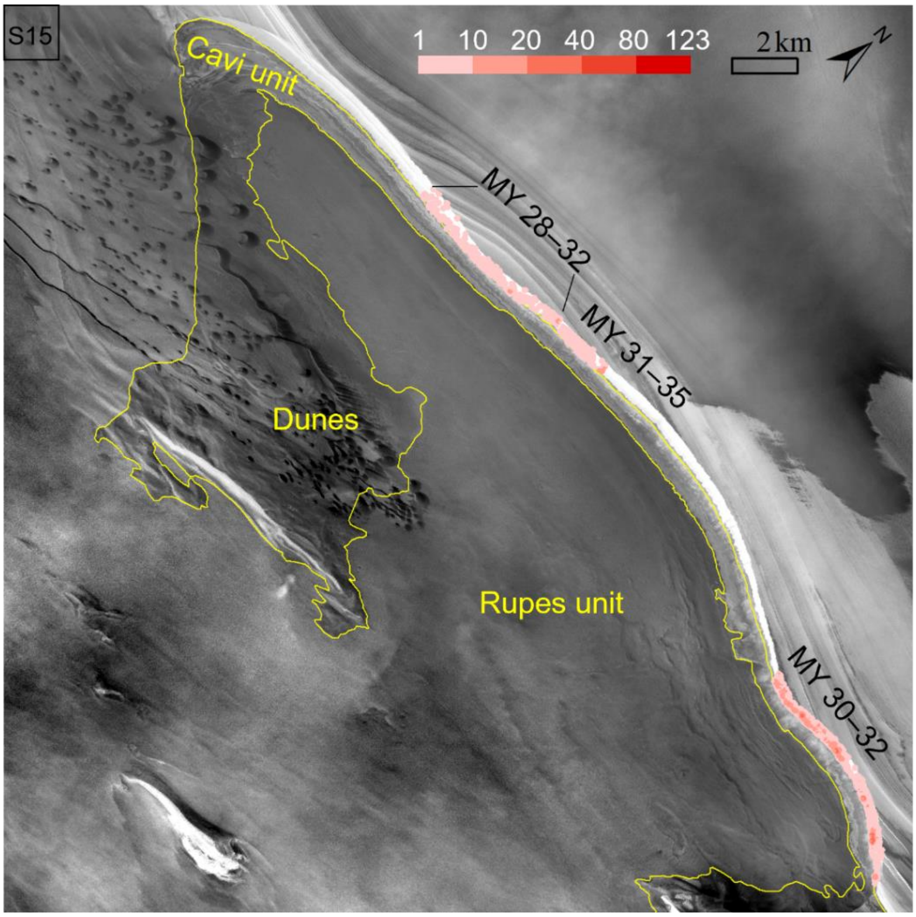

Supplementary Fig. 22 | Scarp S15. Downhill is towards the lower left. The background image is the CTX image P01_001518_2650_XI_85N020W. Credit: background CTX image, NASA/JPL-Caltech/MSSS.

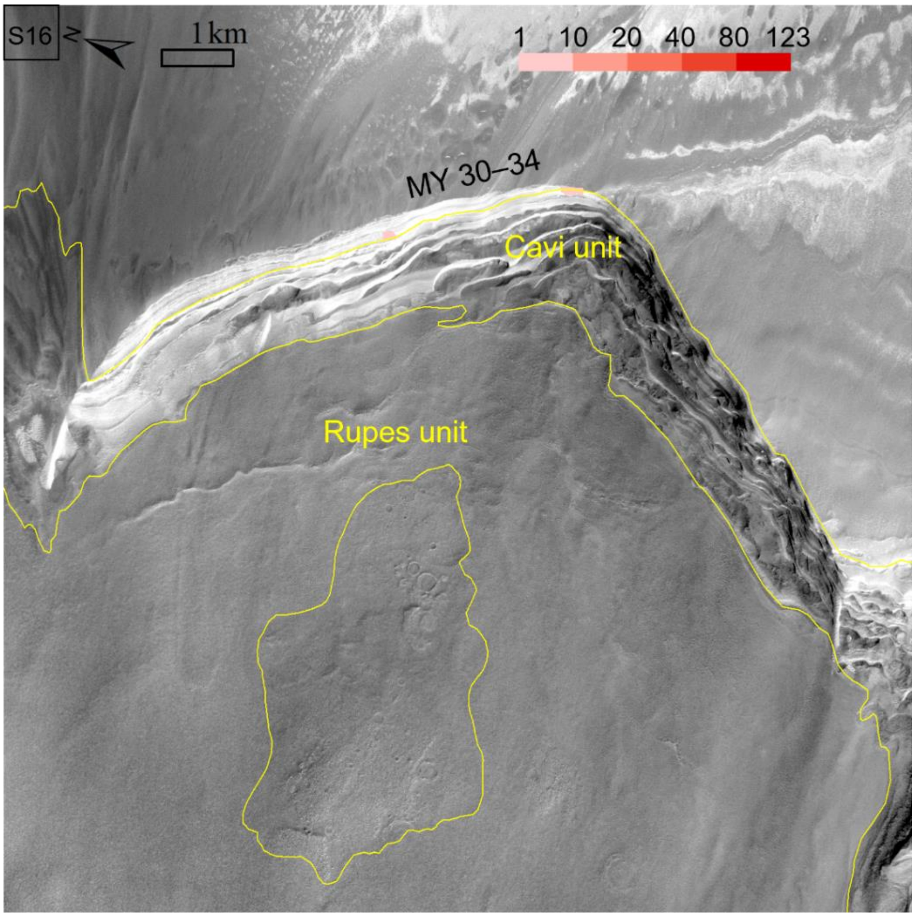

Supplementary Fig. 23 | Scarp S16. Downhill is towards the lower left. The background image is the CTX image F02_036436_2647_XN_84N017W. Credit: background CTX image, NASA/JPL-Caltech/MSSS.

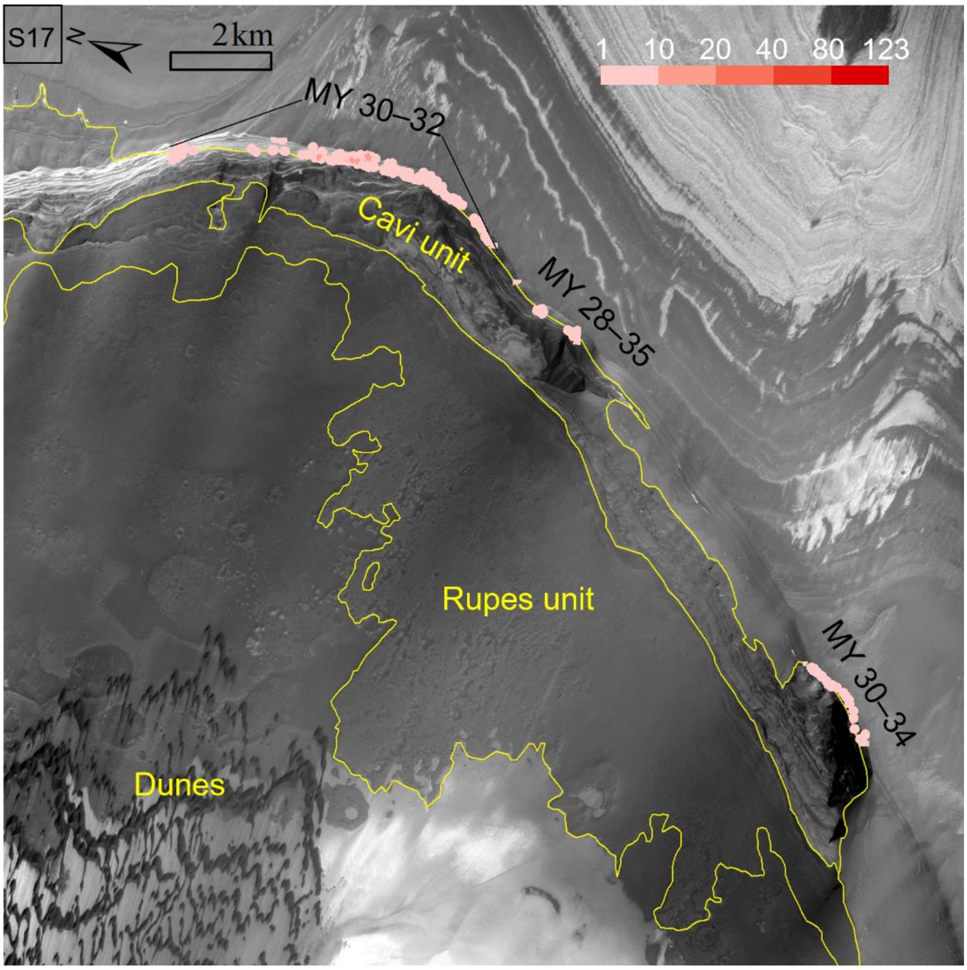

Supplementary Fig. 24 | Scarp S17. Downhill is towards the lower left. The background image is the CTX image F02_036436_2647_XN_84N017W. Credit: background CTX image, NASA/JPL-Caltech/MSSS.

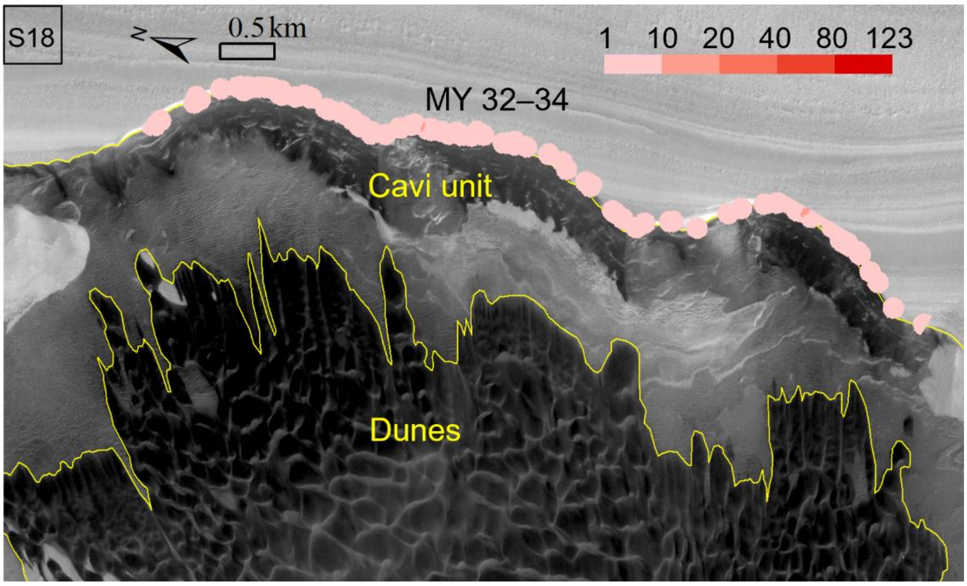

Supplementary Fig. 25 | Scarp S18. Downhill is towards the bottom. The background image is the CTX image D22_035924_2618_XN_81N077W. Credit: background CTX image, NASA/JPL-Caltech/MSSS.

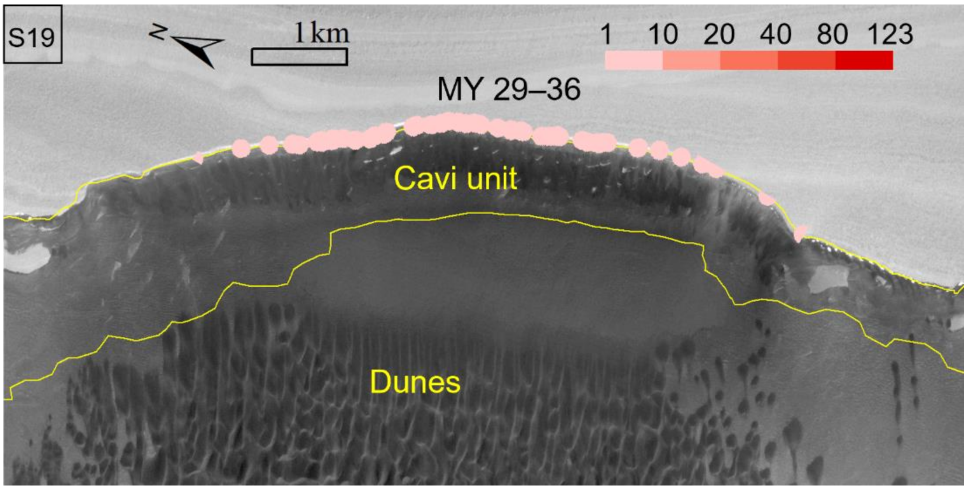

Supplementary Fig. 26 | Scarp S19. Downhill is towards the bottom. The background image is the CTX image N22_071082_2609_XN_80N080W. Credit: background CTX image, NASA/JPL-Caltech/MSSS.

Supplementary Table 1 | Comparison of erosion, retreat and viscous flow rates for the same part of scarp S2 (~12.6 km long) between different studies.

		**Volume** (m^3/martian year)	**Erosion rate** (m^3/martian year per scarp meter)	**Retreat rate** (m/kyr)	**Viscous flow rate** (m/kyr)
**This study**	Active part	16,800.2	1.3	3.6	
	Moderately active part	434.7	0.03	0.1	
	Entire scarp	17,234.9	1.4	2.0	
**Fanara et al.[9]**	NPLD+BU scarp	3,254	0.3	0.2	
**Sori et al.[22]**	NPLD scarp				100–1000

Active part and moderately active part refer to Fig. 3. kyr: 1000 Earth years.

Supplementary Table 2 | List of length, mass wasting volume and erosion rate of each scarp, and their correlation with sand dune, cavi unit, and rupes unit.

**Scarp**	**Length** (km)	**Volume** (m^3/ martian year)	**Erosion rate** (m^3/ martian year per scarp meter)	**Sand dune**	**Cavi unit**	**Rupes unit**
S1	13.8	4305.10	0.31	✓	✓	✓
S2	36.9	32464.78	0.88	✓	✓	✓
S3	22	1500.50	0.07	✓	✓	✓
S4	15.2	809.75	0.05	✓	✓	✓
S5	21.3	6992.35	0.33	✓	✓	✓
S6	12.3	487.49	0.04	✓	✓	✓
S7	7.2	9.95	0.001	✓	✓	✓
S8	7.6	1334.25	0.18	✓	✓	✓
S9	19.9	13198.08	0.66	✓	✓	✕
S10	4.7	57.16	0.01	?	✓	✕
S11	5.9	305.19	0.05	✓	✓	✕
S12	10.5	2100.19	0.20	✓	✓	✕
S13	14.8	721.72	0.05	✓	✓	✕
S14	34.2	7346.94	0.21	✓	✓	✓
S15	15.7	3333.63	0.21	✓	✓	✓
S16	5.7	25.89	0.005	✓	✓	✓
S17	14.6	861.14	0.06	✓	✓	✓
S18	9.6	373.39	0.04	✓	✓	✕
S19	7.7	136.34	0.02	✓	✓	✕
U1				✓	✓	✓
U2				✓	✓	✕
U3				✓	✓	✕
U4				✓	✓	✓

Supplementary Table 3 | HiRISE images used for detecting ice-fragments. The raw HiRISE images were downloaded from the Planetary Data System (https://hirise-pds.lpl.arizona.edu/PDS/).

Scarp	Multi-temporal pair	HiRISE name	Resolution (m)	Mars Year	Date	Incidence angle (°)
S1	p1	ESP_036809_2640	0.25	32	2014-06-03	69
		ESP_054149_2640	0.25	34	2018-02-13	65
	p2	ESP_018981_2640	0.25	30	2010-08-14	66
		ESP_062866_2640	0.25	35	2019-12-25	64
S2	p1	ESP_019047_2640	0.25	30	2010-08-19	67
		ESP_036888_2640	0.25	32	2014-06-09	70
	p2	ESP_019258_2640	0.5	30	2010-09-05	70
		ESP_061943_2645	0.5	35	2019-10-14	59
	p3	ESP_018902_2640	0.25	30	2010-08-08	66
		ESP_026972_2640	0.25	31	2012-04-28	60
	p4	PSP_001378_2640	0.25	28	2006-11-11	67
		ESP_053991_2640	0.25	34	2018-02-01	64
S3	p1	ESP_019548_2650	0.25	30	2010-09-27	75
		ESP_036558_2650	0.25	32	2014-05-15	67
	p2	ESP_019548_2650	0.25	30	2010-09-27	75
		ESP_054004_2650	0.25	34	2018-02-02	65
	p3	ESP_019284_2650	0.25	30	2010-09-07	71
		ESP_054663_2650	0.25	34	2018-03-25	73
S4	p1	ESP_018956_2650	0.25	30	2010-08-12	67
		ESP_036586_2650	0.25	32	2014-05-17	67
	p2	ESP_028015_2650	0.25	31	2012-07-18	70
		ESP_063289_2650	0.25	35	2020-01-27	70
S5	p1	ESP_027791_2655	0.25	31	2012-07-01	67
		ESP_053913_2655	0.25	34	2018-01-26	64
	p2	ESP_018244_2655	0.25	30	2010-06-18	61
		ESP_062340_2655	0.25	35	2019-11-14	61
	p3	ESP_028068_2655	0.25	31	2012-07-22	71
		ESP_063342_2655	0.25	35	2020-01-31	71
S6	p1	PSP_009634_2655	0.25	29	2008-08-16	63
		ESP_054032_2655	0.25	34	2018-02-04	65
S7	p1	ESP_026631_2650	0.25	31	2012-04-01	60
		ESP_053373_2650	0.25	34	2017-12-15	60
S8	p1	ESP_018957_2655	0.25	30	2010-08-12	67
		ESP_036548_2655	0.25	32	2014-05-14	67
S9	p1	ESP_036535_2650	0.25	32	2014-05-13	66
		ESP_053875_2650	0.25	34	2018-01-23	63
	p2	PSP_009648_2650	0.25	29	2008-08-17	62
		ESP_053730_2650	0.25	34	2018-01-12	62
	p3	PSP_009648_2650	0.25	29	2008-08-17	62
		ESP_053875_2650	0.25	34	2018-01-23	63
	p4	ESP_017771_2650	0.25	30	2010-05-12	60
		ESP_036825_2650	0.25	32	2014-06-04	70
S10	p1	ESP_019472_2650	0.25	30	2010-09-21	74
		ESP_036891_2655	0.25	32	2014-06-10	71
S11	p1	ESP_018945_2645	0.25	30	2010-08-11	66
		ESP_062909_2645	0.25	35	2019-12-28	65
S12	p1	ESP_018827_2640	0.25	30	2010-08-02	65
		ESP_054562_2640	0.25	34	2018-03-18	70
	p2	ESP_018115_2640	0.25	30	2010-06-08	60
		ESP_036523_2640	0.25	32	2014-05-12	65

S13	p1	ESP_018959_2635	0.25	30	2010-08-12	66
		ESP_053639_2635	0.25	34	2018-01-05	61
	p2	ESP_036589_2635	0.25	32	2014-05-17	66
		ESP_054549_2635	0.25	34	2018-03-17	70
	p3	ESP_017891_2635	0.25	30	2010-05-21	59
		ESP_063490_2635	0.25	35	2020-02-11	72
S14	p1	PSP_010128_2650	0.25	29	2008-09-23	67
		ESP_036580_2650	0.25	32	2014-05-16	67
	p2	ESP_036435_2650	0.25	32	2014-05-05	65
		ESP_062848_2650	0.25	35	2019-12-23	65
	p3	PSP_009944_2650	0.25	29	2008-09-09	65
		ESP_036488_2650	0.25	32	2014-05-09	66
S15	p1	PSP_001374_2650	0.25	28	2006-11-11	68
		ESP_036634_2650	0.25	32	2014-05-21	68
	p2	ESP_028089_2650	0.25	31	2012-07-24	71
		ESP_062862_2650	0.25	35	2019-12-24	65
	p3	ESP_018911_2650	0.25	30	2010-08-09	66
		ESP_036515_2650	0.25	32	2014-05-11	66
S16	p1	ESP_019044_2650	0.25	30	2010-08-19	69
		ESP_053934_2650	0.25	34	2018-01-28	64
S17	p1	ESP_019043_2645	0.25	30	2010-08-19	68
		ESP_036937_2645	0.25	32	2014-06-13	72
	p2	PSP_001334_2645	0.25	28	2006-11-08	67
		ESP_062572_2645	0.25	35	2019-12-02	62
	p3	ESP_018898_2645	0.25	30	2010-08-08	66
		ESP_054132_2645	0.25	34	2018-02-12	65
S18	p1	ESP_036847_2620	0.25	32	2014-06-06	68
		ESP_053607_2620	0.25	34	2018-01-02	59
S19	p1	PSP_009433_2610	0.25	29	2008-07-31	59
		ESP_071570_2610	0.25	36	2021-11-02	62

Supplementary Method | Height (or depth/thickness) calculation based on cast shadow of the object.

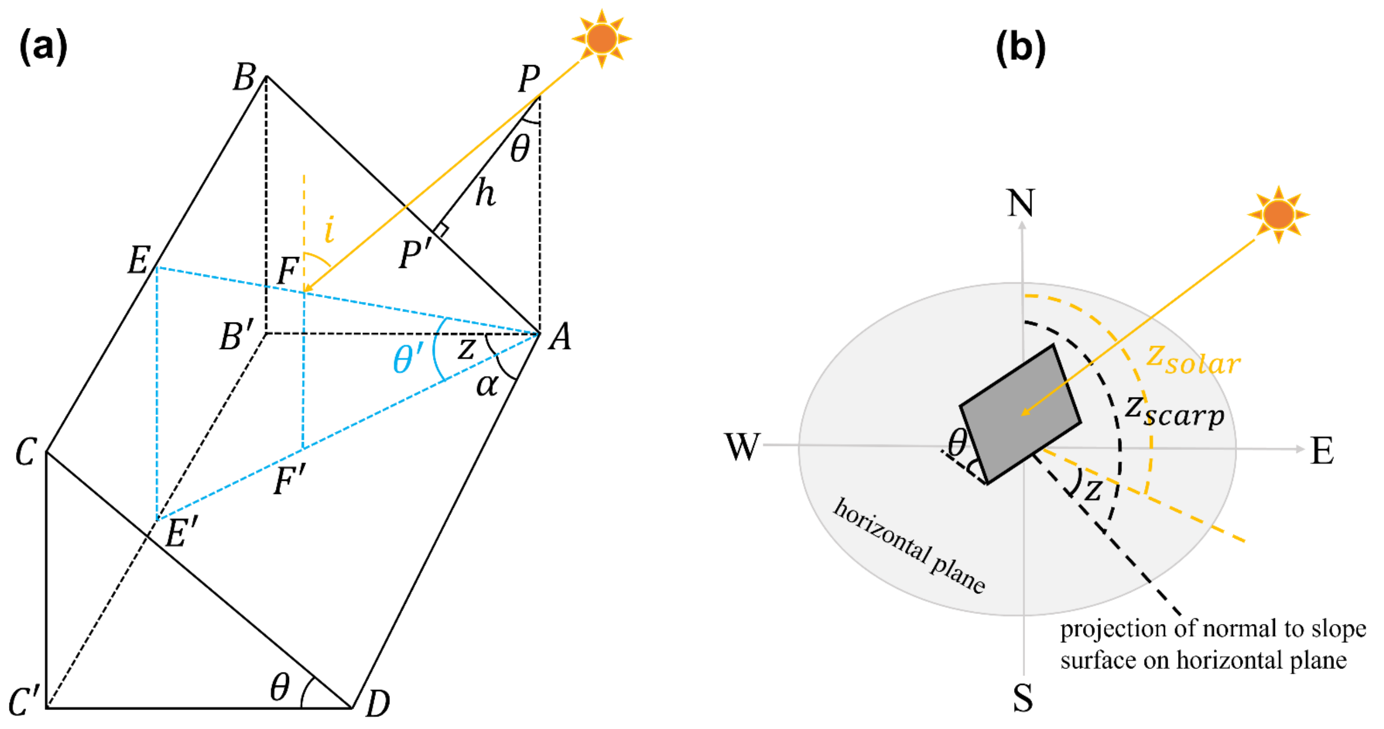

Fig. 1. (a) Graph for the derivation of height from shadow. The height of the object is represented by the line PP', perpendicular to the slope plane $ABCD$. The slope angle is θ. The angle between PP' and PA is thus equal to θ. When the slope is not directly facing the sun, the projection of PP' will be deflected by an aspect z. The new slope angle is θ'. The projection point of P on the slope is F. The angle between the sunlight projection AE' on the horizontal plane and the slope boundary line AD is α. The incidence angle of the sun is i. The cast shadow length measured from the orthorectified image is AF'. (b) The definition of the solar azimuth angle z_{solar} and the slope surface azimuth angle z_{scarp}. Thus, z is the relative azimuth angle of the sun with respect to the slope surface.

Fig. 1a shows the geometric relationship between the height of the object and its cast shadow on the steep scarp. Note that we use the orthorectified HiRISE images, so the influence of the satellite emission angle can be ignored.

In $\triangle ABB'$,

$$\tan(\theta) = BB'/AB' \tag{1}$$

In $\triangle AB'E'$,

$$\cos(z) = AB'/AE' \tag{2}$$

In $\triangle AEE'$,

$$\tan(\theta') = EE'/AE' \tag{3}$$

and,

$$BB' = EE' \tag{4}$$

In most cases, the slope is not directly facing the sun, the new slope angle θ' in the cross section plane AEE' is given by:

$$\tan(\theta') = \tan(\theta)\cos(z) \tag{5}$$

where θ is the slope angle; z is the relative azimuth angle of the sun with respect to the slope surface.

According to Fig. 1b,

$$z = z_{solar} - z_{scarp} \tag{6}$$

where z_{solar} is the solar azimuth angle, z_{scarp} is the slope surface azimuth angle. Both are measured clockwise from the north on the horizontal plane. Angular displacement east of south is negative and west of south is positive. According to the geometric relationship in the Fig. 1a, z can be simplified by:

$$z = 90° - \alpha \tag{7}$$

where α is the angle between the sunlight projection on the horizontal plane and the slope boundary line.

In the right $\triangle APP'$, the height of the object h is given by:

$$h = PA \times \cos(\theta) \tag{8}$$

In the plane $APFF'$,

$$PA = FF' + \frac{AF'}{\tan(i)} \tag{9}$$

$$FF' = AF' \tan(\theta') \tag{10}$$

$$PA = AF' \tan(\theta') + \frac{AF'}{\tan(i)} \tag{11}$$

where AF' is the length of the shadow directly measured from the orthorectified HiRISE image. i is the incidence angle. θ' is the new slope angle.

Therefore, h can be derived as follows:

$$h = AF'[\sin(\theta)\cos(z) + \cos(\theta)\cot(i)] \tag{12}$$
